# Page for the General Public

**DOI:** 10.1055/s-0039-1693039

**Published:** 2019-07-22

**Authors:** Anneke Damberg

**Affiliations:** 1 AORTA Journal Editorial Office, New Haven, Connecticut

The following pages summarize and review this issue's articles for an audience without a background in medicine or research.

1. Syed Usman Bin Mahmood et al: “Acute Type A Aortic Dissection Surgery Performed by Aortic Specialists Improves 2-Year Outcomes”
  It seems intuitive that the more experience a surgeon has in a procedure, the better the results will be. Acute Type A dissection is a life threatening disease that usually needs immediate surgical repair. The authors of this article assessed whether at their institution, the surgeon's level of experience with surgery on the aorta, the body's main artery, had a measurable impact on surgical outcome. The study included 102 patients. They found that experienced surgeons tended to perform more complex and potentially more durable procedures. Patients operated by more experienced aortic surgeons had indeed better survival rates at two years. It therefore seems beneficial to establish special call teams for emergencies of the aorta, provided that the patient can be transferred to an institution with an aortic specialist in a timely fashion.
2. Raphaelle A. Chemtob et al: “Effects of Sex on Early Outcome following Repair of Acute Type A Aortic Dissection: Results from The Nordic Consortium for Acute Type A Aortic Dissection (NORCAAD)”
Previous reports had suggested that women have poorer outcomes after open-heart surgery than men. *Raphaelle A. Chemtob et al* analysed data from a large study on acute aortic dissection, a life threatening disease in which a tear occurs in the wall of the aorta, the body's main artery. The authors found that the 373 women in the study group of 1154 patients were on average older and had more health problems than the men included in the study. On the other hand, disease of the aorta tended to be more extensive in men. With regard to survival, the authors could not find an association between sex and early mortality.
3. Azhar Hussain et al: “Novel Approach to Repairing a Traumatic Aortic Arch Pseudoaneurysm Following a Fall”
  A serious trauma to the chest can cause an injury to the body's main vessel, the aorta. A major bleeding from the aorta is often fatal, but if the leakage is minor, it can lead to a so-called “pseudo-aneurysm”, a blood filled cavity in the chest. If it continues to bleed, this can still be life-threatening. Sometimes, the leakage can be covered with a tubed stent graft prosthesis from the inside, but this was not possible in this case. The alternative option is a complex surgical procedure in which a part of the vessel is replaced with a graft. To avoid a major surgical procedure with vessel replacement, the authors of this article decided to expose the leaking vessel and suture it from the outside. The patient recovered and follow-up imaging showed no further leakage of the aorta.
4. Raymond Pfister et al: “18 FDG-PET—CT to Guide Aortic Arch Replacement in Relapsing Polychondritis”
Polychondritis is a rare autoimmune disease. The patient's immune system attacks structures of the patient's own body. In polychondritis, the immune system mainly attacks cartilage, but it can also lead to inflammation and destruction of blood vessels. *Pfister et al* describe the case of a young woman with polychondritis that had caused inflammation of the aorta, the body's main artery, and the vessels providing blood to the head. The inflammation lead both partly to vessel enlargement and partly to vessel occlusion impeding blood flow to the brain. The vessels needed replacement. To guide the procedure, the authors used an imaging technique called PET-CT. In PET-CT, a radioactive marker is injected into the blood that gathers mainly in tissues with strong metabolic activity, e.g. inflamed tissues. The authors used this imaging technique to differentiate healthy and inflamed tissue and to optimize the procedure.
5. Mario D'Oria et al: “Hypogastric Artery Occlusion with Evoked Potentials Monitoring as Bailout Technique to Assess the Risk of Postoperative Spinal Cord Ischemia”
*D'Oria et al* discuss a case of a patient who had Type B aortic dissection, a disease in which a tear occurs in the wall of the descending part of the aorta, the body's main vessel.
  Dissection can lead to life threatening complications. In this patient, it lead to an obstruction of blood flow to the spinal cord causing paralysis of the legs. Therefore, the authors stabilized the patient's aorta with a tubed stent graft prosthesis they inserted through the groin. The patient recovered well. The patient also had an abnormal enlargement (aneurysm) of a vessel in the basin that can be important for spinal cord blood flow. A procedure to occlude (close) it was scheduled some months later. The authors used a special technique to monitor the patient's nervous system while the patient was under anesthesia to assess if they could occlude the vessel with the aneurysm without risking damage to the spinal cord. The patient recovered without any permanent damage.
6. George S. Georgiadis et al: “Validation of Duplex Ultrasound Graft Surveillance in the Immediate Postoperative Period”
*Georgiadis et al* describe the case of a patient who had an occluded vessel in the groin that was bypassed with a tubed graft prosthesis. After the procedure, everything indicated that the procedure went well and that the graft provided enough blood flow. However, the authors used ultrasound to see if there was sufficient blood flow in the graft, but they could not depict it until 4 days after the procedure. The authors suspect that the inability to depict blood flow in the graft by ultrasound was caused by the fabric of the graft and possibly because of air trapped in the fabric or in the tissue around the graft after the procedure. This observation could be important for vascular surgeons when they want to assess the success of their procedure.
7. Michele Antonello et al: “Transapical Deployment of Thoracic Stent Graft for Ascending Aorta Coronary Bypass Pseudoaneurysm in a Patient with Prosthetic Aortic Valve”
Every surgery can have complications. *Antonello et al* describe the case of a patient who had a rare but serious complication after coronary artery bypass surgery. In this procedure, a blockage in a coronary artery, a vessel that is important to provide blood to the heart muscle, is bypassed with a vessel (a vein) taken from the leg. In this patient, there was a leakage from where the vessel was connected to the aorta, the body's main artery. This leakage led to what is called a “pseudoaneurysm”, a cavity filled with blood. This pseudoaneurysm can lead to life-threatening bleeding and therefore had to be closed. Since the patient was very sick, the authors wanted to avoid a major surgical procedure in which the chest is fully opened up again. Instead, the authors inserted a tubed stent graft prosthesis into the aorta through a small incision in the chest wall and the tip of the heart to occlude the leakage. Since the stent also blocked the bypass vessel, the original coronary vessel was opened with a smaller stent graft to allow sufficient blood flow to the heart muscle. The procedure worked well, the patient recovered and several months later the pseudoaneurysm was fully occluded.

